# In Silico Analysis of the Molecular-Level Impact of SMPD1 Variants on Niemann-Pick Disease Severity

**DOI:** 10.3390/ijms22094516

**Published:** 2021-04-26

**Authors:** François Ancien, Fabrizio Pucci, Marianne Rooman

**Affiliations:** 1 3BIO—Computational Biology and Bioinformatics, Université Libre de Bruxelles, Avenue F. Roosevelt 50, 1050 Brussels, Belgium; fancien@ulb.ac.be (F.A.); fapucci@ulb.ac.be (F.P.); 2 (IB)^2^—Interuniversity Institute of Bioinformatics in Brussels, Boulevard du Triomphe, 1050 Brussels, Belgium

**Keywords:** sphingomyelin phosphodiesterase, Niemann-Pick disease, Parkinson disease, genetic variants, disease severity prediction

## Abstract

Sphingomyelin phosphodiesterase (SMPD1) is a key enzyme in the sphingolipid metabolism. Genetic SMPD1 variants have been related to the Niemann-Pick lysosomal storage disorder, which has different degrees of phenotypic severity ranging from severe symptomatology involving the central nervous system (type A) to milder ones (type B). They have also been linked to neurodegenerative disorders such as Parkinson and Alzheimer. In this paper, we leveraged structural, evolutionary and stability information on SMPD1 to predict and analyze the impact of variants at the molecular level. We developed the SMPD1-ZooM algorithm, which is able to predict with good accuracy whether variants cause Niemann-Pick disease and its phenotypic severity; the predictor is freely available for download. We performed a large-scale analysis of all possible SMPD1 variants, which led us to identify protein regions that are either robust or fragile with respect to amino acid variations, and show the importance of aromatic-involving interactions in SMPD1 function and stability. Our study also revealed a good correlation between SMPD1-ZooM scores and in vitro loss of SMPD1 activity. The understanding of the molecular effects of SMPD1 variants is of crucial importance to improve genetic screening of SMPD1-related disorders and to develop personalized treatments that restore SMPD1 functionality.

## 1. Introduction

Sphingomyelin is a basic constituent of eukaryotic cell membranes, and is primarily found in the myelin sheaths surrounding the nerve cells, which play an important role in the central nervous system [1]. The main enzyme targeting this macromolecule is sphingomyelin phosphodiesterase (SMPD1), which exists in two different forms, the lysosomal form located in the lysosomes and the secretory form located in the extracellular medium [2]. SMPD1 catalyzes the cleavage of sphingomyelin into phosphocholine and ceramide, which is important for sphingomyelin turnover and cell membrane homeostasis. Moreover, as ceramide is a mediator involved in several signaling pathways, SMPD1 also plays an indirect role into downstream signaling processes including cell survival, proliferation, permeability and differentiation [3,4].

Clinical studies have related SMPD1 variants impacting on enzyme activity to the Niemann-Pick disease (NPD) [5,6], an autosomal recessive disorder characterized by a wide variety of pathological symptoms, ranging from jaundice and enlarged abdomen to neurological development delays and death. There are two types of NPD linked to SMPD1, which differ in the severity of their multisystemic clinical manifestations. Patients with type A NPD (NPDA: MIM#257200) suffer from hepatosplenomegaly, pulmonary insufficiency and lack of brain development; they usually die in early childhood [7]. Patients with type B NPD (NPDB: MIM#607616) show varied symptoms mostly linked to viscera, but do not present neurological deficiencies. NPDB is much less severe and patients usually survive up to adulthood. Note that NPD of types C and D are also lysosomal storage diseases but are not linked to SMPD1, and are thus not discussed in this paper.

A promising enzyme replacement therapy for NPDB patients has recently been introduced and consists of the administration of olipudase alfa, a recombinant human acid sphingomyelinase [8,9]. This treatment is currently undergoing clinical trials and preliminary results show significantly alleviated symptoms without major side effects.

Interestingly, growing support for a connection between SMPD1 and a wide series of aging and age-related neurodegenerative diseases is found in the literature [4]; these include Parkinson disease [10,11], Alzheimer disease [12] and major depression [13]. However, the molecular mechanisms that relate the protein variants, their effects on the enzymatic activity and the pathogenic mechanisms leading to these disorders are unknown.

Most annotated SMPD1 variants have been identified by genotyping both alleles of the *smpd1* gene in NPD patients and by searching them for rare variants. This type of analysis is fast and relatively cheap thanks to the advancement of the sequencing technologies, but does not yield a molecular-level explanation of how and why a given variant leads to NPD. Moreover, it is a recessive disease, which makes the association between variants and disease phenotypes difficult to untangle since both alleles have to be considered [14]. In vitro experiments characterizing the effect of variants on SMPD1 are the best approach to gain insights into the pathogenic mechanisms, even though they are expensive and time consuming.

In silico approaches that use 3-dimensional (3D) structural information of the SMPD1 enzyme [6,15,16,17] provide promising alternatives. Indeed, the X-ray structure of SMPD1 has recently been resolved to 2.25 Å resolution [6,16] and allows the detailed study of its conformational stability and activity. SMPD1 basically consists of three domains with different functions. The first is the saposin domain (residues 83–165) formed by four α-helices and stabilized by three disulfide bonds. Previous analyses [6] suggest the role of this domain in the substrate activation of the catalytic reaction. The second domain is a Pro-rich linker region (residues 166–198) that connects the saposin domain to the last domain, which is the catalytic domain (residues 199–611) and contains two 6-stranded β-sheets surrounded by α-helices.

In this paper, we performed bioinformatics analyses based on a series of sequence, structure and annotation information to probe into the molecular effects of SMPD1 variants on catalytic activity, NPD pathogenesis, molecular-level differences between NPDA and NPDB, and connections with Parkinson disease.

## 2. Materials and Methods

### 2.1. Data Collection

We collected the variants in the *smpd1* gene from UniProt [18], ClinVar [19] and the International Niemann-Pick Disease Registry (INPDR) [20], and selected the subset of missense variants. We annotated these variants as disease-causing (D), NPDA-causing (A), NPDB-causing (B), neutral (N) and of unknown significance (U) on the basis of their annotations in the databases from which they were collected. When a variant is present in several databases with different annotations, we followed the following rules:

- If a variant is annotated as U in one database and as D, A, B or N in another database, we used the latter (most informative) annotation.
- If a variant is annotated as D in one database and as A or B in another one, we used the latter (again most informative) annotation.
- In the case of strong conflicting annotations, i.e., when a variant is annotated as N in a database and as D, A or B in another database, we considered the variant as U.
- Variants annotated as NPDA-causing in one database and as NPDB-causing in another database were considered as D.

The way in which we dealt with conflicting annotations is detailed in Supplementary Table S1.

This procedure resulted in a dataset of 309 annotated variants. We then mapped them onto the X-ray structure of SMPD1 (PDB code 5I81) [6]. This led us to reject 43 variants which were not in the sequence section covered by the protein structure. Out of the 266 remaining variants, 40 were annotated as neutral, 151 as disease-causing among which 37 as NPDA and 52 as NPDB, and 75 as of unknown significance. The dataset containing the 40 neutral, 37 NPDA and 52 NPDB variants is called $S_{3cl}$, the set containing the 40 neutral and 151 disease-causing variants is referred to as $S_{2cl}$, and the set of 75 variants of unknown significance is referred to as $S_{\mathrm{VUS}}$. We retrieved the mutant and wild-type allele frequencies from the dbSNP database [21], which were in turn taken from the databases Allele Frequency Aggregator [22], gnomAD [23], ExAC [24] and HapMap [25].

The list of variants in the sets $S_{3cl}$ and $S_{2cl}$, their annotations and their allele frequencies are given in the https://github.com/3BioCompBio/AcidSphingomyelinase repository (accessed on 2 April 2021). Note that we used in this repository and throughout the paper the residue numbering of the 5I81 SMPD1 structure.

### 2.2. Residue Interactions

We focused on disulfide bridges and aromatic-involving interactions in the SMPD1 structure (PDB code 5i81). Disulfide bridges and sulfur-π interactions were obtained using the Protein Interaction Calculator (PIC) [26]. Cation-π interactions between positively charged residues (Arg, Lys) and aromatic residues (Phe, Tyr, Trp, His), amino-π interactions between amino acids carrying a partially charged side chain (Asn, Gln) and aromatic residues (Phe, Tyr, Trp, His), His-π interactions between histidines and aromatic residues (Phe, Tyr, Trp, His), and π-π interactions between two aromatic residues (Phe, Tyr, Trp) were obtained using in-house programs [27,28]. Note the double characteristic of histidines, which are aromatic and sometimes also positively charged.

### 2.3. Features and 2-Class Generic Predictors

To gain insight into the NPD pathogenic mechanisms and set up a 3-class SMPD1-specific deleteriousness predictor, we analyzed a series of sequence-, annotation- and structure-based features listed in Table S2 of Supplementary Material. We first considered the variant deleteriousness scores predicted by three well-known tools: PROVEAN [29], DEOGEN2 [30] and SNPMuSiC [31]. PROVEAN and DEOGEN2 require the amino acid sequence as input while SNPMuSiC requires the 3D protein structure. The PROVEAN predictions are based solely on evolutionary amino acid conservation. DEOGEN2 uses contextual information in addition to evolutionary features, which can be grouped into residue-based, domain-based and protein-based features. As we focus here on a single protein, SMPD1, protein-based features only provide a global shift of the predicted scores. SNPMuSiC scores are obtained as a linear combination of PROVEAN’s evolutionary scores and structural stability scores that we call here SNPMuSiC${}_{\mathrm{SSS}}$:

(1) $$\mathrm{SNPMuSiC}=\alpha_{1}{\mathrm{SNPMuSiC}}_{\mathrm{SSS}}+\alpha_{2}\mathrm{PROVEAN}+\alpha_{3}$$

where $\alpha_{1}$, $\alpha_{2}$ and $\alpha_{3}$ are real values identified in [31]. The SNPMuSiC${}_{\mathrm{SSS}}$ scores correspond to a solvent accessibility-dependent combination of changes in folding free energy caused by mutations (ΔΔW), estimated using various statistical potentials. SNPMuSiC and especially SNPMuSiC${}_{\mathrm{SSS}}$ scores predict variants that are deleterious because they modify (increase or decrease) protein stability. We also considered a predictor of thermodynamic stability changes upon mutations, PoPMuSiC [32], which uses folding free energy contributions that are similar to those of SNPMuSiC${}_{\mathrm{SSS}}$ but are combined in a different way.

Besides these generic deleteriousness and stability predictors that are based on several features and threshold values, we tested a series of single features. These include the changes in folding free energy upon mutation, ΔΔW, computed with the 13 different statistical potentials used in SNPMuSiC${}_{\mathrm{SSS}}$ and in PoPMuSiC, as well as the solvent accessibility of the wild-type residue (Access) and the volume change of the wild-type residue upon mutation (ΔV) [31]. Moreover, the sequence- and domain-dependent features that are included in DEOGEN2 are also considered, i.e., the residue conservation index (EcolCI), the log-odd ratio of the frequency of the wild-type and mutant residues (EvolLOR) [30], the prediction of whether the variant position is part of an early folded region (EarlyF) [33], and the log-odd ratio of deleterious and neutral variant frequencies in PFAM domains (PFAM) [34]. We also considered as features the presence of the variant residue in the saposin domain, in the catalytic domain or in the Pro-rich linker between the catalytic and saposin domains (Saposin, Catalytic, Linker), as well as the spatial distance of the variant residue to the nearest disulfide bond (Disulfide), Zn${}^{2+}$ binding site (Metal), or carbohydrate binding site (Carbohyd). Finally, we considered a series of discrete features describing the polarity, aromaticity and charge of the wild-type or mutant residue (Polarity, Aromatic, Charge).

In total, we considered four predictors and 28 single features. Note that the values of the continuous-valued features *X* were rescaled as follows:

(2) $$X\to \frac{X-\langle X\rangle }{\sigma_{X}}$$

where 〈X〉 and $\sigma_{X}$ are the mean and standard deviation of the distribution of *X* on all 266 variants.

To assess the statistical significance of the ability of these features to differentiate between neutral, NPDA- and NPDB-causing variants, we used an ANOVA F-test for the continuous features and a Chi${}^{2}$ test for the discrete features, in order to estimate the degree of dependency between features and annotations. On the basis of these tests, we selected the features that are statistically significant, with *p*-values ≤ 0.05.

### 2.4. Prediction Method

To combine the selected features into a 3-class prediction model (NPDA-associated, NPDB-associated, neutral), we used a very simple machine learning algorithm, the k-Nearest Neighbour (kNN) algorithm [35,36]. The model estimates the probabilities P(NPDA), P(NPDB) and P(neutral) that a homozygous variant belongs to one of the three classes, based on the class to which the variant’s *k* nearest neighbors belong. In the case of heterozygous variants, we averaged the predicted probabilities from each allele. In a similar way, if multiple variants occur in SMPD1, we computed the final probabilities as the average probabilities over all variants.

The variant is then assigned to the class that has the highest estimated probability value. When two probabilities are equal and higher than the third one, the chosen class is the least deleterious one (N rather than A or B; B rather than A). Note that when only one allele is mutated, the predicted class is always N, in agreement with the recessivity of the disease.

We made the common choice [36] to set the hyperparameter *k* equal to $\sqrt{N}$, where *N* is the number of entries in the training set, in this case 129. To avoid overfitting the results, the predictions were performed using a leave-one-out procedure at the variant position level. This means that, when we predict the effect of a variant at a given position, the training dataset does not contain any variant at the same position.

We evaluated the quality of our 3-class prediction model called SMPD1-ZooM on the basis three different scores: the sensitivity and specificity, defined as the mean of the corresponding quantities for each class, and the balanced accuracy (BACC) score, defined as the mean of the sensitivity and specificity [37]:

(3) $$\mathrm{Sensitivity}=\frac{1}{3}\left(\frac{{\mathrm{TP}}_{\mathrm{NPDA}}}{P_{\mathrm{NPDA}}}+\frac{{\mathrm{TP}}_{\mathrm{NPDB}}}{P_{\mathrm{NPDB}}}+\frac{{\mathrm{TP}}_{\mathrm{neut}}}{P_{\mathrm{neut}}}\right)$$

(4) $$\mathrm{Specificity}=\frac{1}{3}\left(\frac{{\mathrm{TN}}_{\mathrm{NPDA}}}{N_{\mathrm{NPDA}}}+\frac{{\mathrm{TN}}_{\mathrm{NPDB}}}{N_{\mathrm{NPDB}}}+\frac{{\mathrm{TN}}_{\mathrm{Neut}}}{N_{\mathrm{Neut}}}\right)$$

(5) $$\mathrm{BACC}=\frac{\mathrm{Sensitivity}+\mathrm{Specificity}}{2}$$

where P and N represent positives and negatives, respectively, and TP and TN represent true positives and true negatives. The random scores for three classes is 33.3% for sensitivity, 66.7% for specificity and 50.0% for BACC. We also used the area under the receiver operating characteristic curve (AUROC) as performance metric. For three classes, it is obtained by averaging the AUROC of the three binary classifiers (one class versus all).

To estimate the predictor’s performance on two classes (neutral and disease) and compare it with the score of generic deleteriousness predictors, we computed the usual 2-class sensitivity, specificity, BACC and AUROC scores.

### 2.5. Enzymatic Activity

To better assess the molecular impact of variants on SMPD1, we collected from the literature a set of 69 variants for which the relative enzymatic activity (R) has been measured experimentally; they are listed in the https://github.com/3BioCompBio/AcidSphingomyelinase repository (accessed on 2 April 2021). For the variants whose *R*-value is reported in several articles, we considered their mean. Note that we considered here both heterozygous and homozygous variants.

To analyze the relation between the relative enzymatic activity of the variants and their probabilities to be associated with one of the three classes (NPDA, NPDB and neutral), predicted by the SMPD1-ZooM algorithm described in the previous subsections, we computed Pearson’s linear correlation coefficients between each of these three probability values and the *R*-values. Since the relation is expected to be non-linear, we also fitted non-linear functions of the form:

(6) $$P\left(\mathrm{NPDA}\right)=\frac{a_{1}}{a_{1}+R},\;P\left(\mathrm{NPDB}\right)=R\;Exp\left[-a_{2}R+a_{3}\right],\;P\left(\mathrm{Neutral}\right)=a_{4}R+a_{5}R^{2}$$

where $\left(a_{1},\ldots ,a_{5}\right)$ were identified to minimize the root mean square deviation between the experimental points and the fitted curves. These functions have been chosen by trial and error to get root mean square deviations that are as low as possible, with at most two parameters to be fitted.

## 3. Results

### 3.1. Molecular Effect of SMPD1 Variants

**Figure 1:**
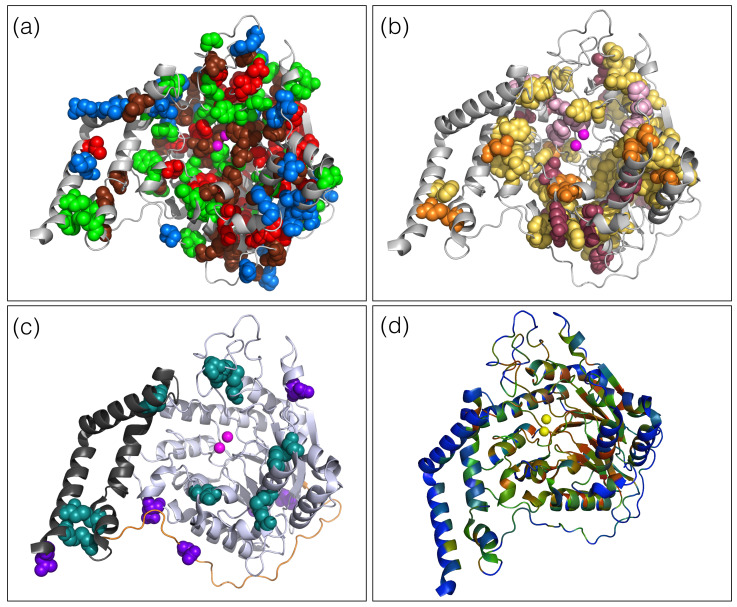
Three-dimensional X-ray structure of SMPD1 (PDB code 5i81). (**a**) NPDA, NPDB, NPD and neutral variant annotations are in red, green, brown and blue spheres, respectively, and Zn ions in magenta; (**b**) Aromatic residues involved in π-π, cation-π, amino-π, sulfur-π and His-π interactions are in yellow spheres, histidines involved in His-π interactions in light pink spheres, Arg, Lys, Gln and Asn involved in cation-π or amino-π interactions in raspberry red spheres, Met and Cys involved in sulfur-π interactions in orange spheres, Zn ions in magenta spheres; (**c**) Saposin domain is in black ribbon, Pro-rich linker domain in orange ribbon, catalytic domain in white-blue ribbon, disulfide bridges in teal spheres, glycosylation sites in purple spheres, Zn ions in magenta spheres; (**d**) SMPD1 is colored according to the 3-state SMPD1-ZooM scores averaged over all 19 possible variants per position, using the RGB color code with red, green and blue representing NPDA, NPDB and neutral predictions, respectively.

To investigate the molecular effect of variants on the SMPD1 structure, we considered its X-ray structure with PDB code 5i81, and mapped all annotated NPDA-, NPDB- and NPD-causing variants and all neutral variants from $S_{3cl}$ and $S_{2cl}$ onto it, as described in Methods. These variants are shown in the SMPD1 structure in Figure 1a.

**Figure 2:**
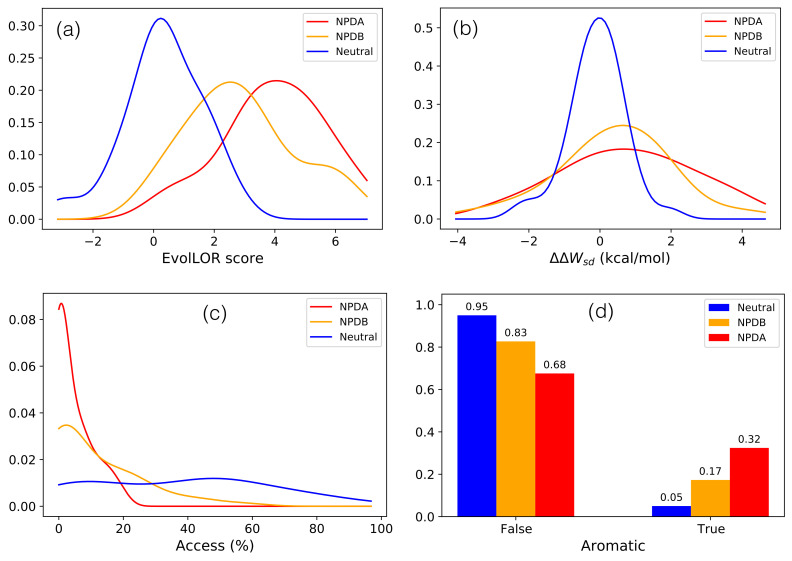
Probability density distributions for neutral (in blue), NPDA-associated (in red) and NPDB-associated variants (in orange). (**a**) EvolLOR evolutionary score; (**b**) $\Delta \Delta W_{sd}$ folding free energy change upon mutation; (**c**) solvent accessibility of the variant residue; (**d**) aromaticity variation upon mutation; “True” means that an aromatic residue is substituted by an non-aromatic residue or that a non-aromatic residue is substituted by an aromatic residue; “False” means that there is no change of aromaticity upon mutation. The ensemble of probability density distributions, for all tested generic predictors and features, are given in Supplementary Figure S1.

Based on these data, we analyzed four deleteriousness and stability predictors and 28 single features, listed in Table S2, for their ability to discriminate between NPDA-causing, NPDB-causing and neutral SMPD1 variants. Several features and predictors are sequence- or evolutionary-based, while others are structure-based and describe protein stability and functional properties. Among these, all four predictors and 10 of the 28 features show a statistically significant discrimination power according to the ANOVA F-test or the Chi${}^{2}$ test (see Mehods). They are listed in Table 1, with some of their associated probability density distributions depicted in Figure 2; the complete series of distributions is shown in Figure S1.

The generic deleterious variant predictors based totally or partly on evolutionary amino acid conservation, i.e., PROVEAN and DEOGEN2, have a good discrimination power (*p*-value <0.001). Variants related to NPDA, known to lead to a high death rate in infancy, are usually introduced at highly conserved positions and are thus likely to have a strong impact on the protein’s structure or function (Figure S1a). NPDB variants are also introduced in conserved protein regions but to a lesser extent compared to NPDA variants. Notably, the two single features that describe evolutionary conservation, EvolCI and EvolLOR, are also able of discriminating the three variant classes (Figure 2a and Figure S1l), which confirms the importance of such type of features.

The stability-based deleteriousness predictor SNPMuSiC${}_{\mathrm{SSS}}$ and the variant stability predictor PoPMuSiC are also able to distinguish between NPBA-, NPDB-associated and neutral variants (*p*-value <0.0001). On the average, NPDA- and NPDB-associated variants have a larger effect on the stability of the protein structure than neutral variants, and this effect is stronger for NPDA than for NPDB variants (Figure S1c,d). Moreover, not only destabilizing variants but also some highly stabilizing variants are NPD causing, as can be seen in PoPMuSiC’s probability density distribution (Figure S1d). Destabilizing variants are expected to lead to local or global changes in the native conformation, whereas the deleterious effect of stabilizing variants is related to the activity-stability trade-off. Indeed, a strong increase in stability often modifies the degree of conformational flexibility, and a higher rigidity usually leads to reduced enzymatic activity. Alternatively, if the stabilizing variant is situated in the active site and has physical-chemical properties that are different from the wild-type, it will be unable to perform the catalytic activity. Note that SNPMuSiC${}_{\mathrm{SSS}}$ predicts as deleterious both highly stabilizing and highly destabilizing variants, so that the distinction between these deleterious variants cannot be directly made using this predictor.

Among the 13 single features that correspond to changes in folding free energy estimated by various statistical potentials, which are included in SNPMuSiC${}_{\mathrm{SSS}}$ and PoPMuSiC, three are found to be statistically significant by themselves: $\Delta \Delta W_{\mathrm{sd}}$, $\Delta \Delta W_{\mathrm{sds}}$ and $\Delta \Delta W_{\mathrm{sad}}$. $\Delta \Delta W_{\mathrm{sd}}$ is based on the propensities to have a given amino acid ‘s’ at a given distance ‘d’ from any other amino acid, $\Delta \Delta W_{\mathrm{sds}}$ on the propensity to have two amino acids ‘s’ separated by a distance ‘d’, and $\Delta \Delta W_{\mathrm{sad}}$ on the propensity to have an amino acid ‘s’ with a solvent accessibility ‘a’ separated by a distance ‘d’ from other amino acids [38]. These three energy functions depend on inter-residue distances and describe tertiary interactions in proteins. Our results thus mean that the modification of specific tertiary interactions is crucial to explain variant deleteriousness in the SMPD1 protein: either stronger stabilization or stronger destabilization of the tertiary structure is observed for NPDA-associated variants, and to a lesser extent for NPDB variants, than for neutral variants (Figure 2b and Figure S1f,g).

The change in aromaticity upon mutation is another statistically significant feature allowing to differentiate between NPDA, NPDB and neutral variants: when a non-aromatic amino acid is substituted by an aromatic amino acid, or conversely, the probability to cause NPDA is significantly larger than the probability to cause NPDB, which is in turn larger than the probability to be neutral, as seen in Figure 2d. To interpret these results, we searched SMPD1 for all aromatic-involving residue-residue interactions (see Methods). We found 18 π-π, 10 cation-π, 7 amino-π, 12 His-π and 14 sulfur-π interactions, listed in Supplementary Table S3. This represents a total of 61 aromatic-involving interactions, which means that 12% of the SMPD1 residues are involved in such an interaction. This is far above the average number found in other proteins. Figure 1b clearly illustrates the abundance of these interactions in the whole protein structure.

It is interesting to emphasize that aromatic-involving interactions and especially π-π interactions are known to confer some flexibility to protein structures [39,40] and to drive liquid-liquid phase transitions [41]. Their abundance makes the SMPD1 structure thus quite a special case with liquid-like interior.

The solvent accessibility of the wild-type residue is an important feature of the SNPMuSiC${}_{\mathrm{SSS}}$ and PoPMuSiC models, which weights their folding free energy terms. It is clearly correlated with variant deleteriousness and stability change, as variants at buried positions have on the average a stronger impact on protein structure than variants introduced in partially buried or solvent exposed regions [31,32,42]. Solvent accessibility is seen here to be moreover able to discriminate between NPDA, NPDB and neutral variants: NPDA-associated variants are usually introduced in totally buried regions (Access ≤ 20%), NPDB-associated variants in totally or partially buried regions (Access ≤ 50%), while neutral variants are almost uniformly distributed over all protein regions, as shown in Figure 2c.

Another selected feature is the spatial distance between variant positions and zinc binding sites; the location of these sites is shown in Figure 1c. The binding to Zn${}^{2+}$ ions is mandatory for the activation of SMPD1 and thus for its enzymatic activity [43]. We observe in Figure S1i that deleterious variants, and especially NPDA-associated ones, tend to be closer to these binding sites. This is probably the result of these variants having a higher probability of impeding or perturbing Zn${}^{2+}$ binding and thus normal SMPD1 functioning.

We also found on the average a smaller distance of NPD variant positions to the glycosylation sites grouped under the symbol Carbohyd, as well as to cysteines involved in disulfide bridges (Figure S1j,k). This can be interpreted as resulting from the structural and functional importance of these features. However, they do not yield a clear differentiation between NPDA and NPDB-associated variants. The localization of disulfide bridges, Zn${}^{2+}$ ions and glycosylation sites in the SMPD1 structure is shown in Figure 1c. Note that there are as many as 8 disulfide bridges (listed in Table S3), spread throughout the structure, which can be viewed as holding together the strongly aromatic liquid-like protein interior.

In summary, the analysis of the selected features show that, while the discrimination between neutral and NPD-associated variants is very accurate, the differentiation between NPDA- and NPDB-associated variants is significantly more challenging. However, for almost all features, the mean value for the NPDB variant class is clearly intermediate between NPDA-associated and neutral variants. This can be taken to mean that the milder symptoms caused by NPDB compared to NPDA are reflected by a milder molecular-level impact of the variants on the SMPD1 protein. Note, moreover, that a strict separation between neutral, NPDA- and NPDB-associated variants is a simplification. Rather, there is a continuous spectrum of phenotypes between benign and NPDA, associated to the existence of intermediate forms of disease [44,45]. The relation between the molecular impact of variants and patient phenotypes is furthermore complicated by the recessivity of NPD, as two disease-causing alleles are needed for the disease phenotype to manifest. The NPD severity and symptoms thus also depend on the combination of both alleles.

### 3.2. Three Class NPD Variant Classifier SMPD1-ZooM

We set up a 3-class NPD-specific deleteriousness predictor called SMPD1-ZooM by combining all predictor scores and individual features listed in Table 1, which are able to distinguish in a statistical significant manner the NPDA-associated, NPDB-associated and neutral variants. For the feature combination, we used a kNN nearest-neighbor algorithm, as described in Methods. The choice of this very simple algorithm was motivated by the smallness of our dataset (129 variants with 3-class annotation) and our desire to avoid introducing parameters and hence to limit overfitting. Moreover the kNN algorithm is well adapted to deal with multiclass problems.

**Figure 3:**
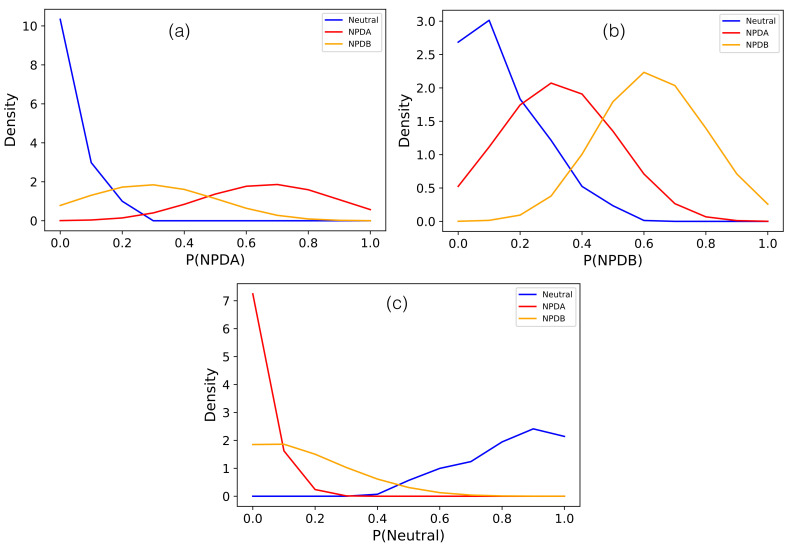
Probability density distributions of (**a**) P(NPDA), (**b**) P(NPDB) and (**c**) P(Neutral) as predicted by the SMPD1-ZooM predictor for neutral (in blue), NPDA-associated (in red) and NPDB-associated variants (in orange).

The capacity of our 3-class predictor to separate NPDA, NPDB and neutral variants is very clear from the probability density distributions in Figure 3. Its performance in leave-one-out cross validation is shown in Table 2 and Table S4: it has a BACC of 79% and an AUROC of 87%. SMPD1-ZooM is freely available for download from https://github.com/3BioCompBio/AcidSphingomyelinase (accessed on 2 April 2021).

As SMPD1-ZooM is currently the only 3-class NPD variant predictor, it cannot be directly compared to other methods. To have nevertheless a basis of comparison, we derived from it, without any optimization process, a 2-class predictor discriminating between neutral and NPD variants, by simply overlooking the difference between NPDA and NPDB predictions (see Methods). This 2-class SMPD1-ZooM predictor has a BACC of 94% and an AUROC as high as 98% in leave-one-out cross validation. As shown in Table 3, it compares favorably with well-known generic deleteriousness predictors such as SIFT [46], PolyPhen-2 [47], MutationAssessor [48], DEOGEN2 [30] and PROVEAN [29]. Note that a specifically trained 2-state NPD-predictor on the whole $S_{2cl}$ set, thus exploiting the additional 62 NPD variants that are are not in $S_{3cl}$ as they have no NPDA or NPDB annotation, is likely to yield even better scores, but this was not our goal. Interestingly, the second best 2-class predictor is the evolution-based PROVEAN algorithm. We wish to underline that PROVEAN’s score is much better on SMPD1 than on other proteins. Indeed, the average PROVEAN BACC score is 72% on a large dataset of mutations in proteins with well resolved structures [31]. This result shows that residue conservation is highly correlated with deleteriousness in SMPD1, much more than in other proteins.

### 3.3. Large-Scale Variant Analysis of SMPD1-ZooM

To better understand SMPD1 robustness with respect to variants, we predicted the impact of every possible amino acid substitution in SMPD1 using the SMPD1-ZooM algorithm described in the previous section. The predictions are available in the https://github.com/3BioCompBio/AcidSphingomyelinase repository (accessed on 2 April 2021). Note that the predicted class of variants from the set S${}_{VUS}$ annotated as of unknown significance, and the NPDA/NPDB class of the variants of $S_{2cl}$ that have no NPDA or NPDB annotation can be found in this repository. Our predictor can therefore play an important role in the management of detected variants whose pathogenic effect is unknown, a common situation in the daily diagnostic routine [49].

**Figure 4:**
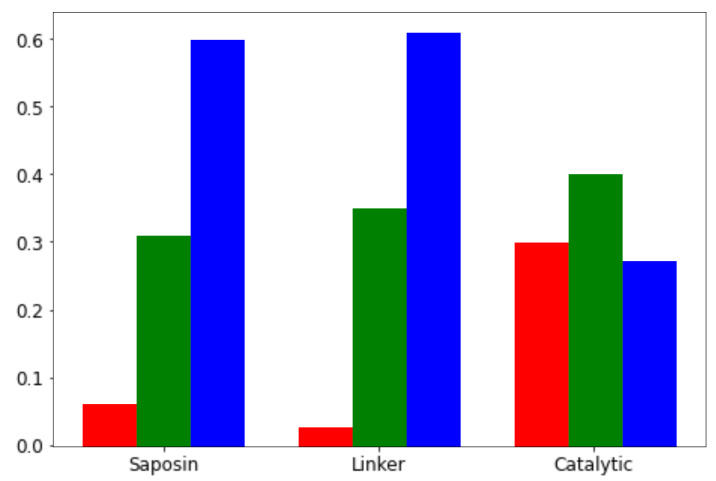
Fraction of variants predicted as neutral (blue), NPDB-associated (green) or NPDA-associated (red) using SMPD1-ZooM, at all sequence positions in the different SMPD1 domains (saposin, proline-rich linker and catalytic domains). The differences between the fractions are all statistically significant according to a proportion Z-test, except the difference between the fractions of neutral variants in the saposin and linker regions, and similarly for the NPDB-associated variants.

Let us first look at the proportion of predicted neutral, NPDB- and NPDA-associated variants in the three domains of SMPD1, i.e., the saposin domain (residues 83–165), the Pro-rich linker (residues 166–198) and the catalytic domain (residues 199–611), represented in Figure 1c. Although belonging to one of these domains did not appear as a statistically significant feature for discriminating the annotated NPDA, NPDB and neutral variants (see Table S2), the large-scale predictions show a statistical significant difference. Indeed, as shown in Figure 4, the fraction of NPDA-associated variants is much larger in the catalytic domain than in the saposin domain, which is in turn larger than in the Pro-rich linker domain. The difference of NPDB-associated variants is more tenuous: it slightly increases from the saposin to the linker and catalytic domains. Our results, which are consistent with previous observations [16], thus suggest that the catalytic domain is the seat of the most serious disease phenotypes.

**Figure 5:**
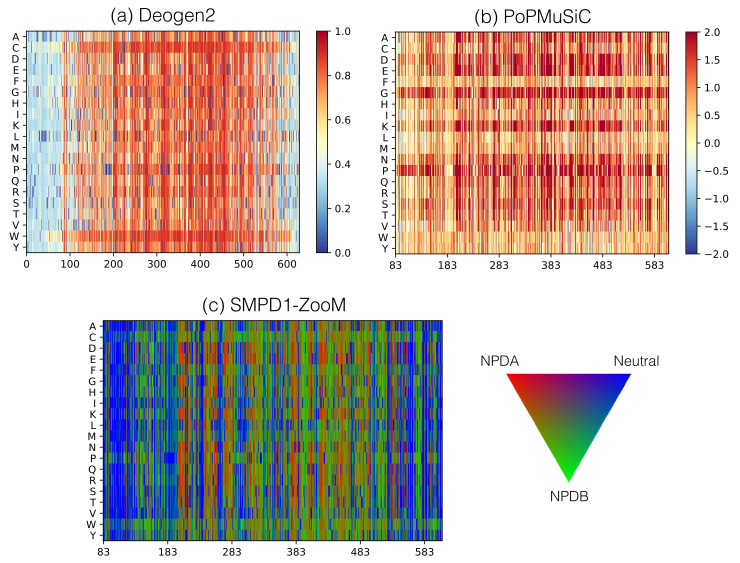
Heatmaps of the scores of all possible amino acid substitutions as a function of the sequence position using (**a**) the 2-state deleteriousness predictor DEOGEN2; (**b**) the ΔΔG (in kcal/mol) predictor PoPMuSiC; (**c**) the 3-state deleteriousness predictor SMPD1-Zoom. The color scale represents: (**a**) red: deleterious and blue: neutral; (**b**) red: destabilizing and blue: stabilizing; (**c**) red: NPDA-associated, green: NPDB-associated, blue: neutral. Note that the sequence on the abscissa is shorter for SMPD1-ZooM and PoPMuSiC than for DEOGEN2 as the former correspond to the X-ray structure and the latter to the full sequence.

More detailed information is obtained by examining the heatmap in Figure 5c representing the SMPD1-ZooM scores of all possible variants along the SMPD1 sequence, and the mapping of average per-residue scores onto the SMPD1 structure in Figure 1d. We clearly see blue areas that are likely to be enriched in neutral variants, such as the solvent exposed residues at the N-terminus of the saposin domain and at the C-terminus of the catalytic domain. Red areas indicating variants likely to lead to NPDA are mainly found close to the catalytic pocket which encompasses residues H206, D278, N318, H457, H459. Green areas are numerous all over the SMPD1 structure, and point to regions where variants are likely to cause the less detrimental NPDB phenotypes. In total, SMPD1-ZooM predicts 64% of all possible variants as NPD-causing, among which 25% as NPDA-associated and 39% as NPDB.

The heatmaps of the generic 2-state deleterious variant predictors give complementary information (Figure 5a and Figure S2a,b). According to DEOGEN2 and SNPMuSiC${}_{\mathrm{SSS}}$, SMPD1 has overall low mutational robustness, which means that a large fraction of its variants have a deleterious effect and lead to a decrease in protein fitness. Indeed, these algorithms predict about 79% and 55% of the all possible variants in SMPD1 as deleterious, respectively, against 37% and 45% in the proteins with known structure of the human proteome. In contrast, PROVEAN predicts roughly the same mutational robustness in SMPD1 and the human proteome (67% against 64%). This indicates that the evolutionary conservation is similar in SMPD1 and other proteins, but that the contextual features drive DEOGEN2 predictions towards lower robustness. Also, the deleterious variants due to stability defects predicted by SNPMuSiC${}_{\mathrm{SSS}}$ are a little more numerous than on average, in accordance with PoPMuSiC’s ΔΔG heatmap (Figure 5b). Indeed, 68% of all possible variants are predicted as destabilizing in SMPD1 and 2% as stabilizing, against 65% destabilizing and 1% stabilizing variants in the human proteome.

The deleteriousness predictors generally agree on the most deleterious variants, even though the ranking differs. But what is systematically conserved is that the most deleterious variants are situated in the catalytic domain (see Tables S5–S7). More precisely, DEOGEN2’s ten most deleterious variants are in the catalytic pocket or close to it (Table S5). This pocket is, as expected, highly conserved as indicated by the PROVEAN scores and any modification in this region is expected to lead to an important loss of SMPD1 activity. These variants are also predicted as deleterious by SNPMuSiC${}_{\mathrm{SSS}}$, and as NPDA-associated by SMPD1-ZooM. The nine most deleterious variants predicted by PROVEAN are Trp residues, known to be highly conserved across evolution in general, and the tenth most deleterious variant is a Cys residue involved in a disulfide bridge (Table S6). The ten most deleterious variants according to SNPMuSiC${}_{\mathrm{SSS}}$ are more diverse (Table S7): Cys residues involved in disulfide bridges, negatively charged residues, glycines, etc.

Finally, the five residues that are the most destabilizing upon mutations according to PoPMuSiC are five aromatic residues (Table S8), which all make π-π and/or sulfur-π interactions (Table S3), except Y367 which makes hydrophobic packing and H-bonds; the spatial environment of these residues is shown in Figure S3. It indicates once again the important role of the network of aromatic-involving interactions in the structural stability and dynamics of SMPD1, especially in the catalytic domain.

Even though the majority of deleterious variants occur in the catalytic domain (Figure 4), the robustness of the saposin domain, which interacts with the lipid membrane, and of the Pro-rich linker, which modulates the relative arrangement of the saposin and catalytic domains [6], is also interesting to study. These domains show an enhanced mutational robustness with respect to the catalytic domain and are less constrained from an evolutionary point of view even though they influence the intracellular localization of sphingomyelinase, its binding to the membrane, its stability and its catalytic activity. Indeed, different variants leading to NPDA or NPDB, i.e., C89H, C92W, L103P, V130A, C131F, L137P and C157R, where the four cysteines are involved in intradomain disulfide bonds, have been reported in the saposin domain. PoPMuSiC predicts all these mutations as destabilizing, with an average ΔΔG of about 1.8 kcal/mol. Note that the saposin domain of a close mammalian homologue of human SMPD1 has been shown to undergo a conformational change that is essential for protein activity by allowing its binding to sphyngomyelin [50]. Thus, variants interfering with this conformational change are expected to lead to a loss of enzymatic activity.

### 3.4. Heterozygous Variant Classification

Given that NPD is a recessive disorder, we have assumed until now that the target variant was present on both alleles when performing predictions. In the general case of heterozygous variants, the probabilities for the patient to have neutral, NPDA or NPDB phenotype were obtained from averaging the probabilities of both alleles, as explained in Section 2.4. To illustrate such predictions, we collected from [10,20,51,52,53] a series of 25 heterozygous variants carried by 18 individuals with annotated phenotypes; 7 of them are affected by NPD and 11 not. These variants are listed in Supplementary Table S9.

We applied SMPD1-ZooM to this set of heterozygous variants, and found that the phenotypes of 17 out of the 18 individuals were predicted correctly, as seen in Supplementary Table S9. Zoom-SMPD1 shows thus a very good accuracy for predicting homozygous but also heterozygous genotypes. Despite the limited number of variants in our heterozygous test set, these results further support the usefulness of our tool for clinical applications.

### 3.5. Focus on SMPD1 Variants in Ashkenazi Jewish Individuals

It is well known that people of Ashkenazi jewish (AJ) ancestry have a significantly increased probability to be affected by NPDA and NPDB compared to the general population [5]. Two deleterious point variants that have been related to this disease are commonly found in AJ individuals: the G → T transversion of nucleotide 1487 occurring at a CpG dinucleotide and resulting in the R496L variant [5], and the T → C transition at nucleotide 905 leading to L302P [54]. Note that other NPD-related variations in the AJ population involve two deletions p.F333Sfs (c.996delC) and p.R610del (c.1829_1831delGCC). Systematic prenatal carrier screenings of SMPD1 variants have thus been implemented in the AJ population [55].

Both variants R496L and L302P are known to be NPDA-associated, and this phenotype is correctly predicted by SMPD1-ZooM (Table 4). Moreover, the three deleteriousness predictors DEOGEN2, PROVEAN and SNPMuSiC${}_{\mathrm{SSS}}$ predict both variants as deleterious. The PoPMuSiC stability change predictor predicts L302P strongly destabilizing, and R496L only marginally so.

**Figure 6:**
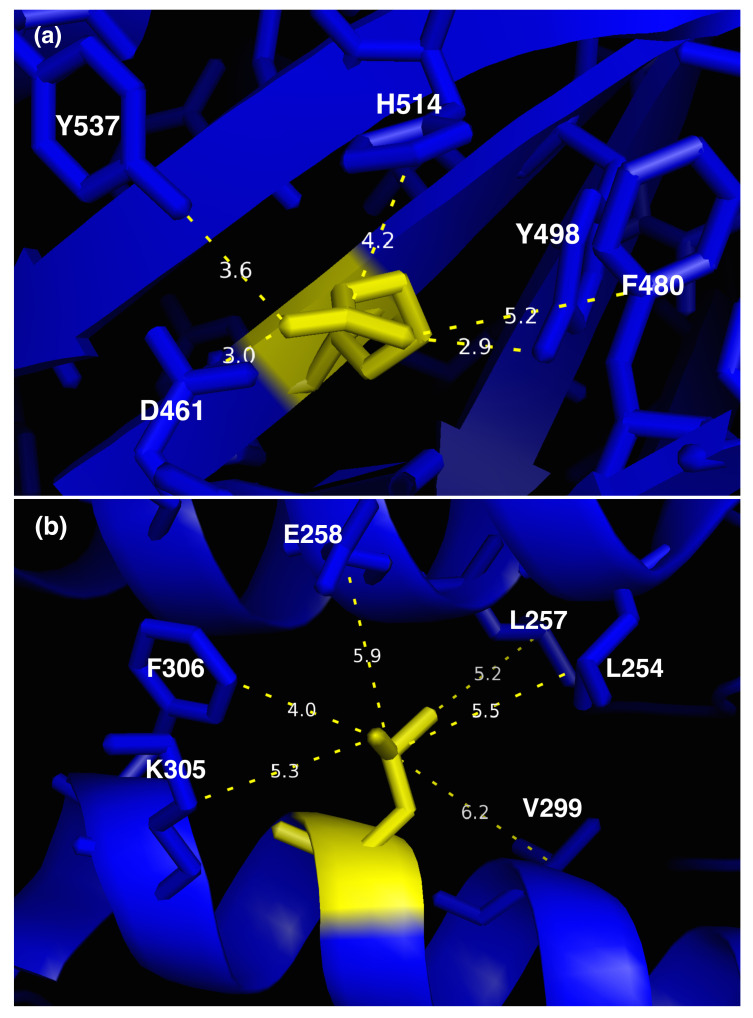
Spatial neighborhood and interactions of residues (**a**) Arg 496 and (**b**) Leu 302 represented in yellow sticks.

The local environment of the two variant residues is shown in Figure 6. R496 forms a salt-bridge with D461, a cation-π interaction with H514 (considered here as aromatic and uncharged) (Table S3) and is surrounded by a cage of aromatic residues Y498, F480 and Y537. Its substitution into Leu destroys these interactions and replaces them by hydrophobic packing with the aromatic residues; the salt bridge is broken, but D461 keeps its anion-π interaction with Y537. This substitution leads to limited destabilization. However, this variant changes the charge distribution inside the core of the catalytic domain, at a distance of 12 Å from the closest residue linked to a Zn${}^{2+}$ ion in the catalytic site. This is expected to perturb the protein’s correct functioning.

The other variant, L302P, is also situated in the catalytic domain, but somewhat further away from the catalytic pocket (15 Å) and closer to the surface. It substitutes a Leu in the middle of an α-helix into a Pro, which is obviously destabilizing and likely to modify locally the structure and flexibility. This explains the strong destabilization predicted by PoPMuSiC. Moreover, this variant modifies the hydrophobic packing with L254, L257, V299 and F306, as Pro is less hydrophobic and has a smaller side chain.

### 3.6. Activity of SMPD1 Variants

**Figure 7:**
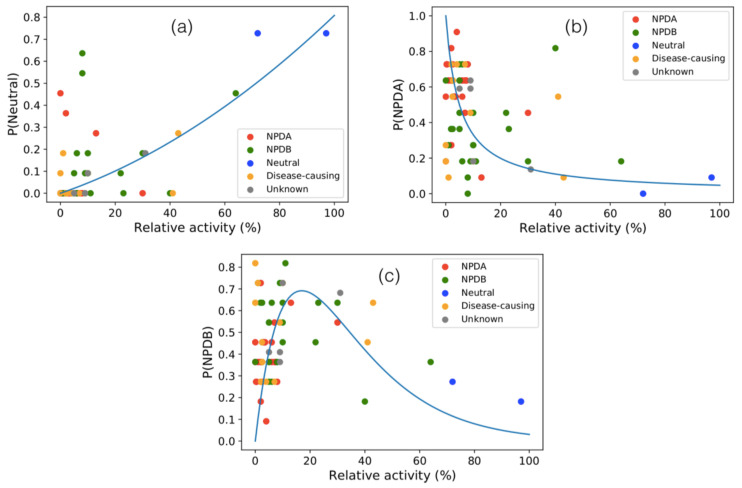
Probabilities of variants to be neutral (**a**), NPDA-associated (**b**) and NPDB-associated (**c**) predicted by SMPD1-ZooM as a function of the relative activity *R* of SMPD1 variants (in %), for the 69 variants for which this activity has been measured. The colors of the points refer to the available clinical annotations. The continuum lines are fitting curves defined in Equation (6).

SMPD1 variants are known to impact on SMPD1 enzymatic properties [20], often making the protein non-functional. We analyzed here a set of 69 variants collected from the literature, which were tested for in vitro activity (see Section 2.5). To analyze the relation between activity and disease phenotypes, we plotted the probabilities of the variants to be NPDA-associated, NPDB-associated or neutral, predicted by SMPD1-ZooM, as a function of their measured relative activity *R* (Figure 7).

Residual enzymatic activity of NPDA-associated variants has been described as almost vanishing, while variants related to NPDB retain a part of it [20]. This is what we also observe from our analysis shown in Figure 7: variants with low activity have higher chance to be NPDA-associated, variants with a residual activity in the 5% to 40% range are likely to lead to NPDB, and variants with activity close to wild-type are usually neutral. The validity of this correlation is supported by the *p*-value of the ANOVA test, which is equal to $5\times 10^{-12}$ for phenotype annotations, and to $2.5\times 10^{-5}$ for SMPD1-ZooM predictions.

Other quantitative measures of this relation are given in Table 5: the linear correlation coefficient between measured activity and predicted probability of disease phenotype is −0.4 for NPDA and 0.6 for neutral; it is non-significant for NPDB because the relation is completely non-linear, as visible in Figure 7c. The root mean square deviation relative to the fitted curves are smallest for the neutral phenotype, i.e., 0.1. Note that we can use the inverse of the non-linear fitting Equation (6) to obtain an estimation of the variants’ activity from the probability of the variants to be in the three classes predicted by SMPD1-ZooM.

### 3.7. SMPD1 Activity and Parkinson Disease

The nice correlation that we have found between predicted disease phenotype probabilities and experimental relative activity of SMPD1 can be important not only in the framework of NPD disease, but also to gain insights into the role of SMPD1 in other disorders. Indeed, while loss-of-function recessive variants in SMPD1 have been primarily related to NPD, recent studies suggest a connection between heterozygous SMPD1 variants and several other diseases such as Parkinson disease (PD) [10,11] and Alzheimer disease (AD) [12]. This is not surprising as SMPD1’s catalytic products are important bioactive lipids involved in a series of signaling pathways of pathophysiological importance.

It is interesting to point out a major difference between SMPD1 variants leading to NPD or PD: the former are characterized by an almost full loss of SMPD1 activity while the latter retain up the 50% of the wild-type activity [10,11]. However, the molecular mechanisms explaining why such variants lead to α-synuclein accumulation, which is the hallmark of PD, is not totally understood. It has been suggested [56] to be linked to the decrease of ceramide, which is one of the enzymatic products of SMPD1. This decrease could lead to a reduction of the aspartate protease cathepsin D, since ceramide specifically binds to it and activates it. Finally, considering that cathepsin D is one of the enzymes devoted to α-synuclein degradation, its decrease leads to the PD-characteristic α-synuclein accumulation.

The analysis of the variants observed in seven independent cohorts of PD patients [10] revealed that only specific SMPD1 variants are associated with PD while others, among which NPD-causing variants, are not. For example, the L302P NPDA-associated variant typical of AJ population (see Section 3.5) is connected to PD as it appears to impair the SMPD1 localization to the lysosome. In general, however, no difference in SMPD1 activity, measured by a mass spectrometry-based assay, is observed between PD patients and controls. Instead, a significant correlation between SMDP1 activity and age of onset (AOO) of PD was found, where patients carrying SMPD1 variants of lower activity have 3.5 to 5.8 year earlier PD onset [10].

Here we focused on the set of individuals of known AOO carrying rare SMPD1 variants described in [10]. We correlated the NPD phenotype probability scores of these variants, predicted by SMPD1-ZooM, with the AOO. As we can see in Figure S5, we do not observe any statistically significant correlation between these two quantities. Moreover, we also do not find any statistically significant correlation between AOO and measured SMPD1 activity, which is in disagreement with [10]; the reason of this disagreement is that patients without SMPD1 variants were included in this earlier study.

These results suggests that *only some* heterozygous SMPD1 variants that impact on protein activity lead to an early AOO in the carrier individuals [11], while others do not seem to show a clear trend or *even lead* to a later AOO. An example of this counterintuitive behavior is the rare variant W391G that impacts on protein activity and, when homozygous, leads to NPDA with mild to severe neural involvement [57]. While we would expect that heterozygous W391G leads to an early AOO, PD patients carrying this variant in the cohort of patients analyzed in [10] have a late AOO of more than 70 years on the average. Since PD is a complex disease, we certainly cannot expect that its AOO depends uniquely on SMPD1 activity and fitness. The relations obtained have thus to be considered in a larger context where also other important PD genes and their variants, such as GAB and LRKK2, have to be investigated.

The analysis in this section shows that the SMPD1-ZooM predictor can be used not only in the framework of Niemann-Pick disease, but also as a tool to gain insights into the role of SMPD1 in other disorders such as PD.

## 4. Conclusions

We leveraged structural and stability information to better understand the variants’ effects on SMPD1 at the molecular level, and how these variants can lead to non-functional proteins. Pathogenic SMPD1 variants are mainly involved in NPD, a lysosomal storage disease characterized by a birth prevalence of about 0.5/100,000. Our in silico study led to the development of the SMPD-ZooM algorithm that accurately predicts not only the deleteriousness of SMPD1 variants but also the disease severity. Indeed, this predictor is able to identify which variants are associated to NPDA, the severe form of the disorder characterized by lack of brain development often leading to death in the early childhood, and which variants are associated to NPDB, the mild form characterized by less severe symptoms. The SMPD1-ZooM algorithm is available as a user-friendly program to be used by geneticists and clinicians.

The analysis of all informative features including stability features such as the folding free energy change upon mutations, evolutionary information such as the conservation index in homologous proteins, and structural features such as the solvent accessibility of the variant residue, allowed the identification of regions that are either extremely or poorly mutationally robust. It improved our understanding of the central role of certain interactions such as aromatic-involving interactions and disulfide bridges in maintaining the structural and functional properties of SMPD1.

Furthermore, we found a good correlation between the measured relative catalytic activity of SMPD1 variants and their probability predicted by SMPD1-ZooM to be NPDA-associated, NPDB or neutral. The quantification of this non-trivial relation is an important step which makes it possible to estimate the loss or gain of enzymatic activity for a given SMPD1 variant. SMPD1 is also known to play a role in other disorders such as PD, and a correlation between the relative activity of SMPD1 variants and the age of PD onset of the individuals carrying these variants has been recently described. We explored this hypothesis, but found no statistically significant correlation between the probabilities predicted by SMPD1-ZooM and the AAO. Note that PD is a complex disease and such a relation has to be put in a more complex context involving also the fitness of other PD-related proteins.

In order to deepen the current analysis on SMPD1 and its link with different disorders, more attention has to be given to the role of variants’ combinations. Indeed, the phenotypic effect of a given variant depends on the full genetic background including all other rare and common variants in the protein sequence of the individual.

We would like to stress that SMPD1 is a promising drug target, as its dysregulation is related to a large number of yet other diseases that range from major depression to Alzheimer disease and from atherosclerosis to various cancers. In this context, our analysis of the mutational robustness of SMPD1 can contribute to elucidate the molecular mechanisms involved in these diseases. Even though in vitro functional analyses remain necessary to confirm in silico SDMP1-Zoom predictions, our analysis is a first step, at the clinical level, towards better screening of patients for SDMP1-related diseases and, at the biotechnological level, towards the optimization of drug design approaches aimed at restoring SMPD1’s normal functionality. We would like to emphasize that the characterization of genetic variants in clinical practice is gaining more and more importance in the management of affected patients with the advent of the era of pharmacogenomics and personalized medicine [58,59]. 

## Figures and Tables

**Table 1:** List of generic predictors and features that can statistically significantly distinguish neutral, NPDA-causing and NPDB-causing variants. The full list of predictors and features that have been considered are given in Supplementary Table S2.

Feature/Predictor	Description	Data Type	*p*-Value
PROVEAN	Deleterious variant predictor	Sequence/Evolution	<0.001
DEOGEN2	Deleterious variant predictor	Sequence/Evolution/Context	<0.001
SNPMuSiC${}_{\mathrm{SSS}}$	Deleterious variant predictor	Structure/Stability	<0.001
PoPMuSiC	Variant stability change predictor	Structure/Stability	<0.001
$\Delta \Delta W_{\mathrm{sd}}$	Change in sd folding free energy	Structure/Stability	0.046
$\Delta \Delta W_{\mathrm{sds}}$	Change in sds folding free energy	Structure/Stability	<0.001
$\Delta \Delta W_{\mathrm{sad}}$	Change in sad folding free energy	Structure/Stability	0.018
Access	Variant solvent accessibility	Structure	<0.001
Metal	Variant’s distance from Zn ions	Structure	<0.001
Carbohyd	Variant’s distance from glycosylation site	Structure	0.002
Disulfide	Variant’s distance from disulfide bridge	Structure	0.009
EvolCI	Evolutionary conservation index	Sequence/Evolution	<0.001
EvolLOR	Evolutionary Log-odd ratio	Sequence/Evolution	<0.001
Aromatic	Aromaticity variation	Sequence	0.017

**Table 2:** Scores in leave-one-out cross validation of the SMPD1-specific variant classifier SMPD1-ZooM on three classes (neutral, NPDA- and NPDB-associated).

SMPD1-ZooM (3-Class)			
Sensitivity	Specificity	BACC	AUROC
73.3%	86.0%	79.7%	86.9%

**Table 3:** Two-class classification scores of commonly used deleteriousness predictors and the 2-class version of our 3-state SMPD1-specific predictor SMPD1-ZooM on the $S_{2cl}$ dataset. Highest scores are in bold.

Method	Sensitivity	Specificity	BACC	AUROC
SMPD1-ZooM (2-class)	89.4%	**97.5**%	**93.5**%	**97.8**%
SNPMuSiC [31]	85.4%	87.5%	86.5%	94.0%
SNPMuSiC${}_{\mathrm{SSS}}$ [31]	82.8%	67.5%	75.1%	86.2%
DEOGEN2 [30]	96.0%	80.0%	88.0%	96.9%
SIFT [46]	60.9%	95.0%	78.0%	88.8%
PolyPhen2 [47]	**96.7**%	85.0%	90.8%	96.6%
PROVEAN [29]	87.4%	**97.5**%	92.5%	96.1%
MutationAssessor [48]	86.8%	82.5%	84.6%	93.8%

**Table 4:** Molecular-effect predictions of the missense variants commonly found in populations of AJ ancestry by various deleteriousness predictors used in this study, as well as by the ΔΔG predictor PoPMuSiC. Red values mean deleterious for all predictors but PoPMuSiC for which it means destabilizing; the ΔΔG value in black indicate only a marginal effect on stability.

Predictor	L302P	R496L
SMPD1-ZooM (3-class)	NPDA	NPDA
DEOGEN2	0.88	0.83
PROVEAN	−4.39	−6.65
SNPMuSiC${}_{\mathrm{SSS}}$	0.25	0.24
PoPMuSiC	3.93 kcal/mol	0.24 kcal/mol

**Table 5:** Linear correlation coefficient ($r_{\mathrm{linear}}$) between the measured relative activity of SMPD1 variants and the probability of variants to be in one of the three classes predicted by SMPD1-ZooM, with the *p*-value in parentheses; root mean square deviation ($\sigma_{\mathrm{nonlinear}}$) between the probability values and the fitted curves depicted in Figure 7.

	$r_{\mathrm{linear}}$	$\sigma_{\mathrm{nonlinear}}$
P(NDPA)	−0.38 (*p* =0.001)	0.30
P(NPDB)	−0.11 (*p* =0.38)	0.26
P(Neutral)	0.61 (*p* $\;<10^{-5}$)	0.14

## Data Availability

All the data and predictions that we generated as well as the SMPD1-ZooM algorithm are freely available for download from our GitHub repository (https://github.com/3BioCompBio/AcidSphingomyelinase accessed on 2 April 2021).

## Supplementary Materials

The following are available online at https://www.mdpi.com/article/10.3390/ijms22094516/s1. Table S1: Resolution of annotation conflicts; Table S2: Generic predictors and individual features; Figure S1: Density distribution of predictors and features; Table S3: Residue-residue interactions; Table S4: SMPD1-ZooM predictor; Figure S2: Heatmaps; Table S5: Large-scale analysis of mutational robustness (DEOGEN2); Table S6: Large-scale analysis of mutational robustness (PROVEAN); Table S7: Large-scale analysis of mutational robustness (SNPMuSiC); Table S8: Large-scale analysis of mutational robustness (PoPMuSiC); Figure S3: Large-scale analysis of mutational robustness (3D environment); Table S9: Prediction for heterozygous variants; Figure S4: SMPD1 and Parkinson disease.
