# Supplementary material for: In Silico Analysis of the Molecular-Level Impact of SMPD1 Variants on Niemann-Pick Disease Severity
Source: Int J Mol Sci. 2021 Apr 26;22(9):4516. doi: 10.3390/ijms22094516 (PMC8123603; doi:10.3390/ijms22094516)
Supplement: Supplementary file 1 [file ijms-22-04516-s001.zip › ijms-1137197-supplementary.pdf]

# Supplementary Materials:
# In silico analysis of the molecular-level impact of SMPD1 variants on Niemann-Pick disease severity.

**François Ancien, Fabrizio Pucci and Marianne Rooman**

1. Resolution of annotation conflicts
2. Generic predictors and individual features
3. Density distribution of predictors and features
4. Residue-residue interactions
5. SMPD1-ZooM predictor
6. Heatmaps
7. Large-scale analysis of mutational robustness
8. Prediction for heterozygous variants
9. SMPD1 and Parkinson disease

## 1. Resolution of annotation conflicts

| Annotation Database 1 | Annotation Database 2 | Final annotation |
|---|---|---|
| Unknown | Disease-causing<br>NPDA-causing<br>NPDB-causing<br>Neutral | Disease-causing<br>NPDA-causing<br>NPDB-causing<br>Neutral |
| Disease-causing | NPDA-causing<br>NPDB-causing<br>Neutral | NPDA-causing<br>NPDB-causing<br>Unknown |
| Neutral | NPDA-causing<br>NPDB-causing | Unknown<br>Unknown |
| NPDA-causing | NPDB-causing | Disease-causing |

**Table S1.** Final variant annotation considered in case the annotations differ between two databases.

## 2. Generic predictors and individual features

| Name | Based on | Description | Type | p-value |
|---|---|---|---|---|
| PROVEAN | Sequence | PROVEAN [1] deleterious variant prediction score | Continuous | <0.001 |
| DEOGEN2 | Sequence | DEOGEN2 [2] deleterious variant prediction score | Continuous | <0.001 |
| SNPMuSiC$_{SSS}$ | Structure | Structural stability score of the deleterious variant predictor SNPMuSiC [3] | Continuous | <0.001 |
| PoPMuSiC | Structure | Thermodynamic stability change upon mutation predicted by PoPMuSiC [4] | Continuous | <0.001 |
| $\Delta\Delta W_{st}$ | Structure | Residue-torsion angle potential of SNPMuSiC [3] | Continuous | 0.244 |
| $\Delta\Delta W_{stt}$ | Structure | Residue-torsion angle-torsion angle potential of SNPMuSiC [3] | Continuous | 0.217 |
| $\Delta\Delta W_{sst}$ | Structure | Residue-residue-torsion angle potential of SNPMuSiC [3] | Continuous | 0.277 |
| $\Delta\Delta W_{sa}$ | Structure | Residue-solvent accessibility potential of SNPMuSiC [3] | Continuous | 0.333 |
| $\Delta\Delta W_{saa}$ | Structure | Residue-accessibility-accessibility potential of SNPMuSiC [3] | Continuous | 0.252 |
| $\Delta\Delta W_{ssa}$ | Structure | Residue-residue-solvent accessibility potential of SNPMuSiC [3] | Continuous | 0.469 |
| $\Delta\Delta W_{sta}$ | Structure | Residue-torsion angle-solvent accessibility potential of SNPMuSiC [3] | Continuous | 0.542 |
| $\Delta\Delta W_{sd}$ | Structure | Residue-distance potential of SNPMuSiC [3] | Continuous | 0.046 |
| $\Delta\Delta W_{sds}$ | Structure | Residue-distance-residue potential of SNPMuSiC [3] | Continuous | <0.001 |
| $\Delta\Delta W_{sad}$ | Structure | Residue-solvent accessibility-distance potential of SNPMuSiC [3] | Continuous | 0.018 |
| $\Delta\Delta W_{sadsa}$ | Structure | Residue-accessibility-distance-residue-accessibility potential of SNPMuSiC [3] | Continuous | 0.316 |
| $\Delta\Delta W_{std}$ | Structure | Residue-torsion angle-distance potential of SNPMuSiC [3] | Continuous | 0.946 |
| $\Delta\Delta W_{stdst}$ | Structure | Residue-torsion-distance-residue-torsion potential of SNPMuSiC [3] | Continuous | 0.816 |
| Access | Structure | Solvent accessibility (in %) of the mutated residue [3] | Continuous | <0.001 |
| $\Delta V$ | Sequence | Difference in volume between wild-type and mutant residues [3] | Continuous | 0.488 |
| EvolCI | Sequence | Evolutionary conservation index [2] | Continuous | <0.001 |
| EvolLOR | Sequence | Log-odd ratio of observing the wild-type with respect to the mutant residue [2] | Continuous | <0.001 |
| EarlyF | Sequence | Prediction of whether the mutated residue is in an early-folded region [2] | Continuous | 0.161 |
| PFAM | Sequence | Log-odd ratio of deleterious/neutral variant frequency in PFAM domains [2] | Continuous | 0.333 |
| Saposin | Sequence | 1 if the variant is in the saposin domain, 0 otherwise | Discrete | 0.216 |
| Linker | Sequence | 1 if the variant is in the Pro-rich linker between the two domains, 0 otherwise | Discrete | 0.426 |
| Catalytic | Sequence | 1 if the variant is in the catalytic domain, 0 otherwise | Discrete | 0.701 |
| Disulfide | Structure | Distance between mutated residue and closest disulfide bond | Continuous | 0.009 |
| Metal | Structure | Distance between mutated residue and closest metal binding site | Continuous | <0.001 |
| Carbohyd | Structure | Distance between mutated residue and closest carbohydrate binding residue | Continuous | 0.002 |
| Aromatic | Sequence | 1 if wild-type or mutant residue is aromatic but not both, 0 otherwise | Discrete | 0.017 |
| Polarity | Sequence | 1 if wild-type and mutant residues have the same polarity, 0 otherwise | Discrete | 0.349 |
| Charge | Sequence | 1 if wild-type and mutant residues do not have the same charge, 0 otherwise | Discrete | 0.469 |

**Table S2.** List of generic predictors and individual features analyzed to set up a SMPD1-specific deleteriousness predictor. Residues TYR, PHE and TRP are aromatic, ARG, LYS and HIS are taken as positively charged, ASP and GLU as negatively charged, GLN, ASN, SER, THR and TYR as non-charged polar, and ALA, ILE, LEU, MET, PHE, VAL, PRO, GLY, CYS and TRP as non-polar, following the classification of [5]. Spatial distances were computed between the geometric centers of the amino acid side chains. The saposin, Pro-rich linker and catalytic domains encompass residues 83-165, 166-198 and 199-611, respectively [6]. Annotations were taken from UniProt [7] and PFAM [8]. P-values indicate the capacity to distinguish NPDA-, NPDB-associated and neutral variants using the ANOVA F-test and Chi$^2$ for continuous and discrete features, respectively.

## 3. Density distribution of predictors and features

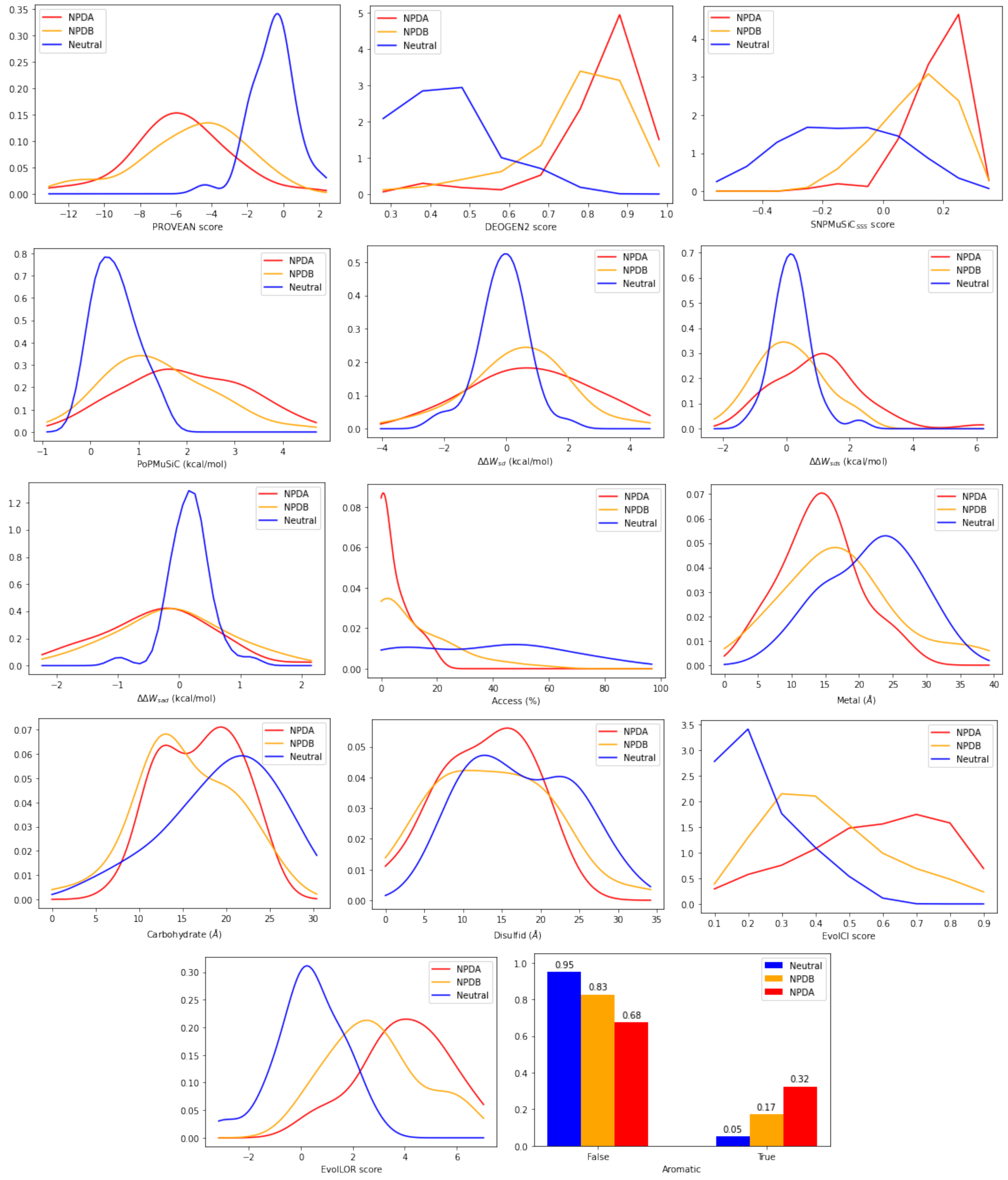


**Figure S1.** Probability density distributions for all generic predictors and features that are statistically significant for discriminating between neutral (in blue), NPDA-associated (in red) and NPDB-associated (in orange) variants, which are described in Table 1 of the main text.

## 4. Residue-residue interactions

| Interaction type | amino acid (position) | |
|---|---|---|
| $\pi$-$\pi$ interactions | F (A 138) | F (A 390) |
| | W (A 174) | W (A 435) |
| | F (A 203) | F (A 454) |
| | Y (A 243) | W (A 244) |
| | Y (A 274) | F (A 454) |
| | F (A 366) | W (A 405) |
| | F (A 390) | W (A 391) |
| | W (A 435) | Y (A 439) |
| | Y (A 440) | F (A 466) |
| | F (A 454) | Y (A 498) |
| | F (A 463) | F (A 570) |
| | F (A 480) | Y (A 498) |
| | F (A 480) | W (A 553) |
| | Y (A 495) | Y (A 517) |
| | Y (A 495) | W (A 533) |
| | Y (A 537) | Y (A 543) |
| | F (A 567) | W (A 571) |
| | W (A 571) | Y (A 574) |
| His-$\pi$ interactions | H (A 208) | H (A 282) |
| | H (A 208) | H (A 319) |
| | H (A 282) | H (A 319) |
| | H (A 282) | Y (A 488) |
| | H (A 286) | W (A 285) |
| | H (A 514) | F (A 480) |
| | H (A 514) | Y (A 498) |
| | H (A 575) | W (A 571) |
| | H (A 578) | Y (A 247) |
| | H (A 575) | W (A 571) |
| | H (A 575) | Y (A 574) |
| | H (A 578) | Y (A 247) |

| Interaction type | amino acid (position) | |
|---|---|---|
| cation-$\pi$ and amino-$\pi$ interactions | R (A 255) | Y (A 243) |
| | R (A 255) | W (A 244) |
| | R (A 289) | W (A 340) |
| | R (A 294) | H (A 211) |
| | K (A 305) | F (A 306) |
| | N (A 381) | F (A 384) |
| | N (A 383) | F (A 327) |
| | Q (A 402) | F (A 366) |
| | K (A 433) | W (A 437) |
| | N (A 438) | W (A 174) |
| | R (A 445) | Y (A 446) |
| | Q (A 453) | F (A 466) |
| | R (A 496) | H (A 514) |
| | N (A 503) | Y (A 372) |
| | Q (A 596) | F (A 463) |
| | R (A 600) | W (A 437) |
| | R (A 600) | Y (A 440) |
| Sulfur-$\pi$ interactions | C (A 92) | F (A 96) |
| | M (A 142) | F (A 390) |
| | C (A 157) | F (A 96) |
| | M (A 272) | Y (A 313) |
| | M (A 272) | Y (A 372) |
| | M (A 345) | W (A 349) |
| | M (A 382) | W (A 435) |
| | M (A 382) | F (A 455) |
| | C (A 385) | W (A 435) |
| | C (A 431) | W (A 435) |
| | M (A 560) | F (A 570) |
| | C (A 584) | F (A 567) |
| | C (A 584) | W (A 571) |
| | C (A 588) | W (A 571) |

| Interaction type | amino acid (position) | |
|---|---|---|
| Disulfide bonds | C (A 89) | C (A 165) |
| | C (A 92) | C (A 157) |
| | C (A 120) | C (A 131) |
| | C (A 221) | C (A 226) |
| | C (A 227) | C (A 250) |
| | C (A 385) | C (A 431) |
| | C (A 584) | C (A 588) |
| | C (A 594) | C (A 607) |

**Table S3.** Aromatic-involving interactions $\pi$-$\pi$, His-$\pi$, cation-$\pi$, amino-$\pi$ and sulfur-$\pi$ (see Methods for their precise definition) and disulfide bridges.

## 5. SMPD1-ZooM predictor

| | **Prediction** | | | |
|---|---|---|---|---|
| **Clinical data** | Neutral | NPDA | NPDB | Total |
| Neutral | 39 | 0 | 1 | 40 |
| NPDA | 2 | 24 | 11 | 37 |
| NPDB | 6 | 16 | 30 | 52 |
| Total | 47 | 40 | 42 | 129 |

**Table S4.** Confusion matrix of the 3-state SMPD1-ZooM prediction model in leave-one-out cross validation.

## 6. Heatmaps

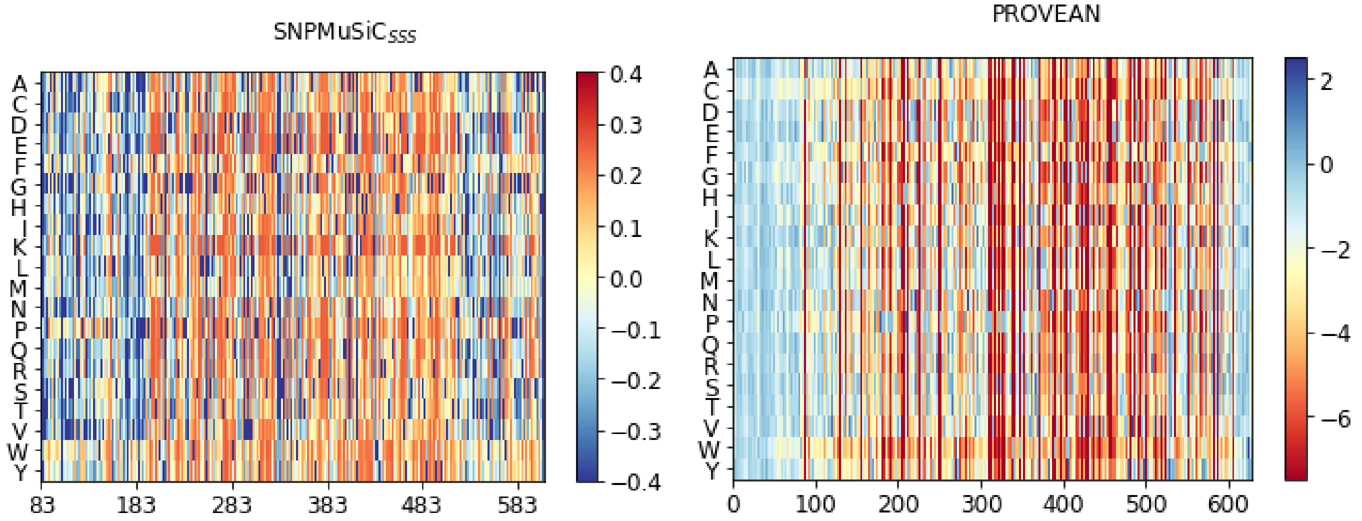


**Figure S2.** Heatmaps of the scores of all possible amino acid substitutions as a function of the sequence position using the 2-state generic deleteriousness predictors SNPMuSiC$_{SSS}$ (a) and PROVEAN (b). The color scale is shown to the right of the figures, with red representing deleterious and blue neutral variants. Deleterious/neutral variant prediction thresholds are 0 for SNPMuSiC$_{SSS}$ and -2.5 for PROVEAN. Note that the sequence on the abscissa is shorter for SNPMuSiC$_{SSS}$ than for PROVEAN as the former corresponds to the X-ray structure and the latter to the full sequence.

## 7. Large-scale analysis of mutational robustness

| Residue | DEOGEN2 | METAL (Å) | PROVEAN | $SNPMuSiC_{SSS}$ | PoPMuSiC | SMPD1-ZooM |
|---|---|---|---|---|---|---|
| G277 | 0.92 | 4.7 | -7.6 | 0.23 | 2.05 | NPDA |
| H425 | 0.92 | 0.0 | -8.8 | 0.20 | 1.31 | NPDA |
| D206 | 0.92 | 0.0 | -7.3 | 0.24 | 0.96 | NPDA |
| H457 | 0.92 | 0.0 | -8.7 | 0.03 | 0.92 | NPDA |
| H208 | 0.92 | 0.0 | -8.9 | 0.24 | 1.98 | NPDA |
| D278 | 0.92 | 0.0 | -7.2 | 0.24 | 0.19 | NPDA |
| G456 | 0.91 | 5.2 | -7.7 | 0.22 | 1.13 | NPDA |
| G245 | 0.91 | 13.2 | -7.4 | 0.25 | 2.52 | NPDA |
| G317 | 0.91 | 3.6 | -8.1 | 0.22 | 1.50 | NPDA |
| D251 | 0.91 | 4.8 | -7.0 | 0.23 | 0.08 | NPDA |

**Table S5.** The ten SMPD1 residues that are the least mutationally robust as predicted by DEOGEN2, ranked in decreasing order. The scores of PROVEAN and $SNPMuSiC_{SSS}$, PoPMuSiC's $\Delta\Delta G$ values (in kcal/mol) and the SMPD1-ZooM predictions are also given, as well as the distance (in Å) between the residue of interest and the closest residue linked to Zn ions in the catalytic site (column METAL). The scores per position are obtained as the mean over the 19 possible amino acid variants at that position. Values in red correspond to disease-causing positions. DEOGEN2 scores range from 0 to 1, with mutations predicted as deleterious having a score above 0.5; the PROVEAN scores mean deleterious if $< -2.5$, the $SNPMuSiC_{SSS}$ scores mean deleterious if $>0$, PoPMuSiC $\Delta\Delta G$ values are stabilizing if $< 0.5$ kcal/mol and destabilizing if $> 0.5$ kcal/mol.

| Position | PROVEAN | DEOGEN2 | $SNPMuSiC_{SSS}$ | PoPMuSiC | METAL (Å) | SMPD1-ZooM |
|---|---|---|---|---|---|---|
| W340 | -13.11 | 0.87 | 0.08 | 2.35 | 14.8 | NPDA |
| W352 | -12.51 | 0.88 | 0.17 | 2.79 | 17.2 | NPDA |
| W405 | -12.51 | 0.91 | 0.01 | 1.79 | 18.2 | NPDA |
| W533 | -12.38 | 0.87 | 0.18 | 2.98 | 15.5 | NPDA |
| W285 | -12.36 | 0.85 | 0.20 | 2.95 | 13.8 | NPDA |
| W349 | -12.22 | 0.90 | 0.20 | 3.17 | 13.1 | NPDA |
| W391 | -12.16 | 0.82 | 0.21 | 3.25 | 14.7 | NPDA |
| W174 | -11.96 | 0.83 | 0.12 | 2.38 | 19.6 | NPDA |
| W553 | -11.06 | 0.85 | 0.18 | 2.93 | 19.6 | NPDA |
| C165 | -9.62 | 0.88 | 0.14 | 1.64 | 36.8 | NPDA |

**Table S6.** The ten positions in SMPD1 with the most deleterious mean PROVEAN scores. See legend of Table S5 for further details.

| Position | $SNPMuSiC_{SSS}$ | DEOGEN2 | PROVEAN | PoPMuSiC | METAL (Å) | SMPD1-ZooM |
|---|---|---|---|---|---|---|
| G245 | 0.25 | 0.91 | -7.43 | 2.52 | 13.2 | NPDA |
| C250 | 0.25 | 0.91 | -10.19 | 3.21 | 11.5 | NPDA |
| E464 | 0.25 | 0.74 | -4.72 | -0.27 | 11.1 | NPDB |
| C227 | 0.24 | 0.89 | -10.19 | 2.85 | 13.5 | NPDA |
| C385 | 0.24 | 0.83 | -9.21 | 2.70 | 9.7 | NPDA |
| P493 | 0.24 | 0.88 | -8.07 | 0.62 | 11.05 | NPDA |
| H208 | 0.24 | 0.92 | -8.86 | 1.98 | 0.0 | NPDA |
| D206 | 0.24 | 0.92 | -7.31 | 0.96 | 0.0 | NPDA |
| D278 | 0.24 | 0.91 | -7.16 | 0.19 | 0.0 | NPDA |
| G365 | 0.24 | 0.88 | -4.12 | 2.67 | 12.6 | NPDA |

**Table S7.** The ten positions in the SMPD1 structure (PDB code 5i81) with the most deleterious mean $SNPMuSiC_{SSS}$ scores. See legend of Table S5 for further details.

| Position | PoPMuSiC | METAL (Å) | DEOGEN2 | PROVEAN | SNPMuSiC$_{SSS}$ | SMPD1-ZooM |
|---|---|---|---|---|---|---|
| F454 | 3.63 | 13.5 | 0.89 | -6.94 | 0.19 | NPDA |
| Y367 | 3.47 | 14.1 | 0.90 | -7.58 | 0.21 | NPDA |
| Y495 | 3.44 | 13.4 | 0.84 | -7.76 | 0.16 | NPDA |
| W435 | 3.38 | 13.4 | 0.82 | -11.92 | 0.20 | NPDA |
| Y498 | 3.33 | 14.1 | 0.86 | -7.72 | 0.20 | NPDA |
| D415 | -0.24 | 30.6 | 0.67 | -1.78 | -0.40 | Neutral |
| E464 | -0.27 | 11.0 | 0.74 | -4.72 | 0.25 | NPDB |
| L233 | -0.28 | 24.4 | 0.49 | -0.73 | -0.45 | Neutral |
| G265 | -0.32 | 22.5 | 0.63 | -1.60 | -0.52 | Neutral |
| P90 | -0.84 | 42.5 | 0.51 | -1.95 | -0.20 | Neutral |

**Table S8.** The five positions in the SMPD1 structure (PDB code 5i81) with the most destabilizing mean $\Delta\Delta G$ values (in kcal/mol) predicted by PoPMuSiC, followed by the five positions with most stabilizing $\Delta\Delta G$ values. See legend of Table S5 for further details.

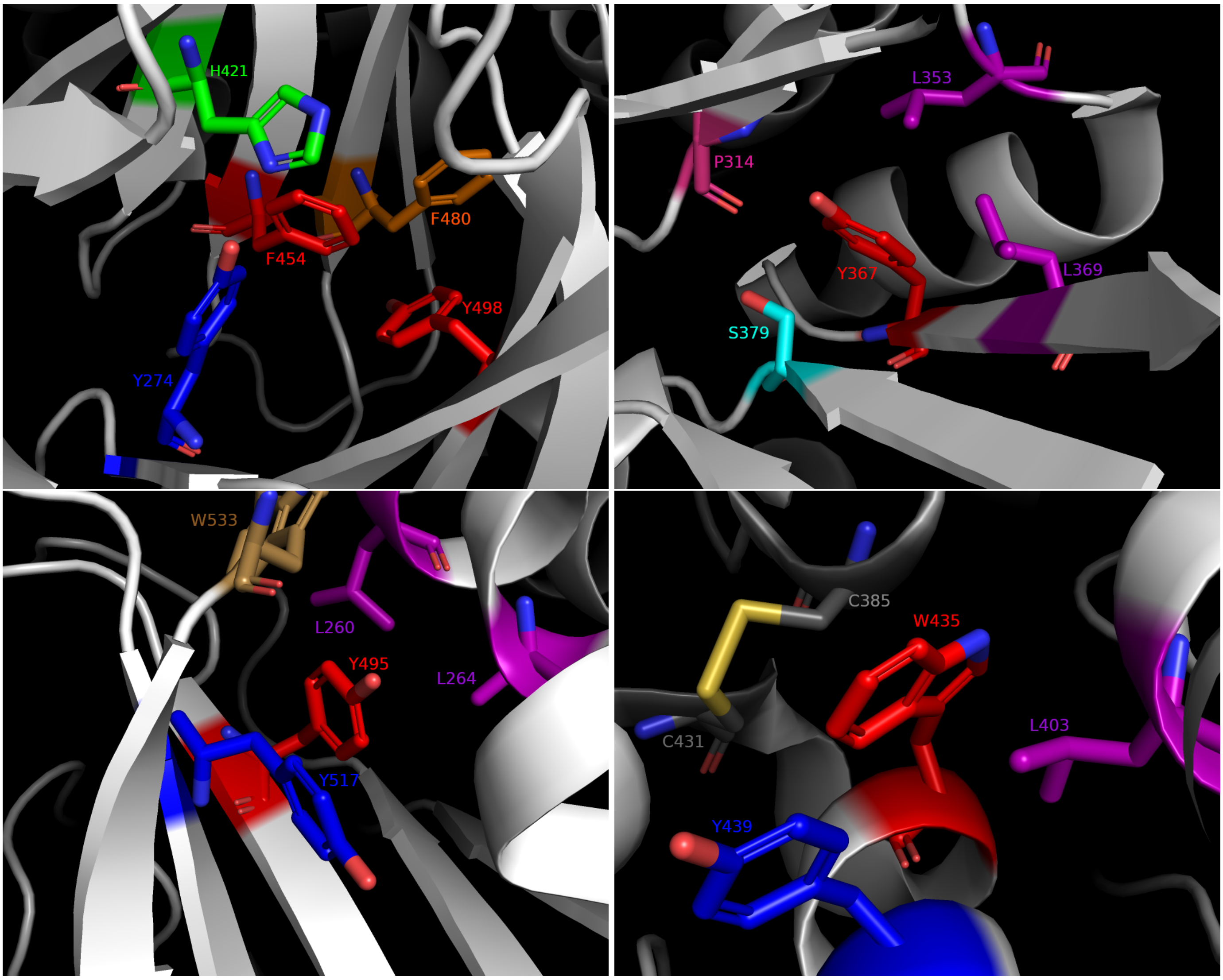


**Figure S3.** Representation of the spatial environment in the SMPD1 structure of the five residues which are predicted as the most destabilizing by PoPMuSiC (see Table S5): F454 and Y498 (a), Y367 (b), Y495 (c) and W435 (d). These residues are in red, phenylalanines in orange, histidines in green, tyrosines in blue, leucines in purple, tryptophans in dark yellow and cysteines in gray.

## 8. Prediction for heterozygotes

| Genotype | 1st allele ZooM score | 1st allele annotation (Homozygous case) | 2nd allele ZooM score | 2nd allele annotation (Homozygous case) | Mean ZooM score | Prediction | Annotation |
|---|---|---|---|---|---|---|---|
| F463S / P475L | N: 0.0%<br>A: 90.9%<br>B: 9.1% | A | N: 0.0%<br>A: 63.6%<br>B: 36.4% | A | N: 0.0%<br>A: 77.3%<br>B: 22.7% | A | A |
| A281T / W244C | N: 9.1%<br>A: 18.2%<br>B: 72.7% | B | N: 0.0%<br>A: 54.5%<br>B: 45.5% | B | N: 4.5%<br>A: 36.4%<br>B: 59.1% | B | B |
| G245S / R600P | N: 0.0%<br>A: 63.6%<br>B: 36.4% | A | N: 0.0%<br>A: 45.5%<br>B: 54.5% | B | N: 0.0%<br>A: 54.5%<br>B: 45.5% | A | B |
| T256I / R600H | N: 0.0%<br>A: 54.5%<br>B: 45.5% | B | N: 27.3%<br>A: 9.1%<br>B: 63.6% | A | N: 13.6%<br>A: 31.8%<br>B: 54.5% | B | B |
| R289H / H575D | N: 36.4%<br>A: 9.1%<br>B: 54.5% | U | N: 0.0%<br>A: 18.2%<br>B: 81.8% | U | N: 18.2%<br>A: 13.6%<br>B: 68.2% | B | B |
| I176N / Q596R | N: 0.0%<br>A: 27.3%<br>B: 72.7% | B | N: 72.7%<br>A: 9.1%<br>B: 18.2% | B | N: 36.4%<br>A: 18.2%<br>B: 45.5% | B | B |
| C431R / Y517C | N: 0.0%<br>A: 63.6%<br>B: 36.4% | D | N: 0.0%<br>A: 54.5%<br>B: 45.5% | A | N: 0.0%<br>A: 59.0%<br>B: 41.0% | A | A |
| W391G/- | N: 0.0%<br>A: 72.7%<br>B: 27.3% | B | N: 100.0 %<br>A: 0.0 %<br>B: 0.0% | N | N: 50.0%<br>A: 36.4%<br>B: 13.6% | N | N |
| G528A/- | N: 90.9%<br>A: 0.0%<br>B: 9.1% | U | N: 100.0%<br>A: 0.0%<br>B: 0.0% | N | N: 95.4%<br>A: 0.0%<br>B: 4.6% | N | N |
| E515V/- | N: 9.1%<br>A: 18.2%<br>B: 72.7% | B | N: 100.0%<br>A: 0.0%<br>B: 0.0% | N | N: 54.6%<br>A: 9.1%<br>B: 36.4% | N | N |
| C157R/- | N: 0.0%<br>A: 63.6%<br>B: 36.4% | B | N: 100.0%<br>A: 0.0%<br>B: 0.0% | N | N: 50.0%<br>A: 31.8%<br>B: 18.2% | N | N |
| R387C/- | N: 9.1%<br>A: 9.1%<br>B: 81.8% | U | N: 100.0%<br>A: 0.0%<br>B: 0.0% | N | N: 54.6%<br>A: 4.6%<br>B: 40.9% | N | N |
| R376H/- | N: 0.0%<br>A: 54.5%<br>B: 45.5% | D | N: 100.0%<br>A: 0.0%<br>B: 0.0% | N | N: 50.0%<br>A: 27.3%<br>B: 22.8% | N | N |
| V112M/- | N:100.0%<br>A: 0.0%<br>B: 0.0% | U | N: 100.0%<br>A: 0.0%<br>B: 0.0% | N | N: 100.0%<br>A: 0.0%<br>B: 0.0% | N | N |
| G268S/- | N: 81.8%<br>A: 0.0%<br>B: 18.2% | U | N: 100.0%<br>A: 0.0%<br>B: 0.0% | N | N: 90.9%<br>A: 0.0%<br>B: 9.1% | N | N |
| R540Q/- | N: 72.7%<br>A: 0.0%<br>B: 27.3% | N | N: 100.0%<br>A: 0.0%<br>B: 0.0% | N | N: 86.4%<br>A: 0.0%<br>B: 13.7% | N | N |
| L254P/- | N: 54.5%<br>A: 9.1%<br>B: 36.4% | U | N: 100.0%<br>A: 0.0%<br>B: 0.0% | N | N: 77.3%<br>A: 4.6%<br>B: 18.2% | N | N |
| G506R/- | N: 72.7%<br>A: 0.0%<br>B: 27.3% | N | N: 100.0%<br>A: 0.0%<br>B: 0.0% | N | N: 86.4%<br>A: 0.0%<br>B: 13.7% | N | N |

**Table S9.** Annotations and SMPD1-ZooM predictions for variants in 18 heterozygous individuals.

## 9. SMPD1 and Parkinson disease

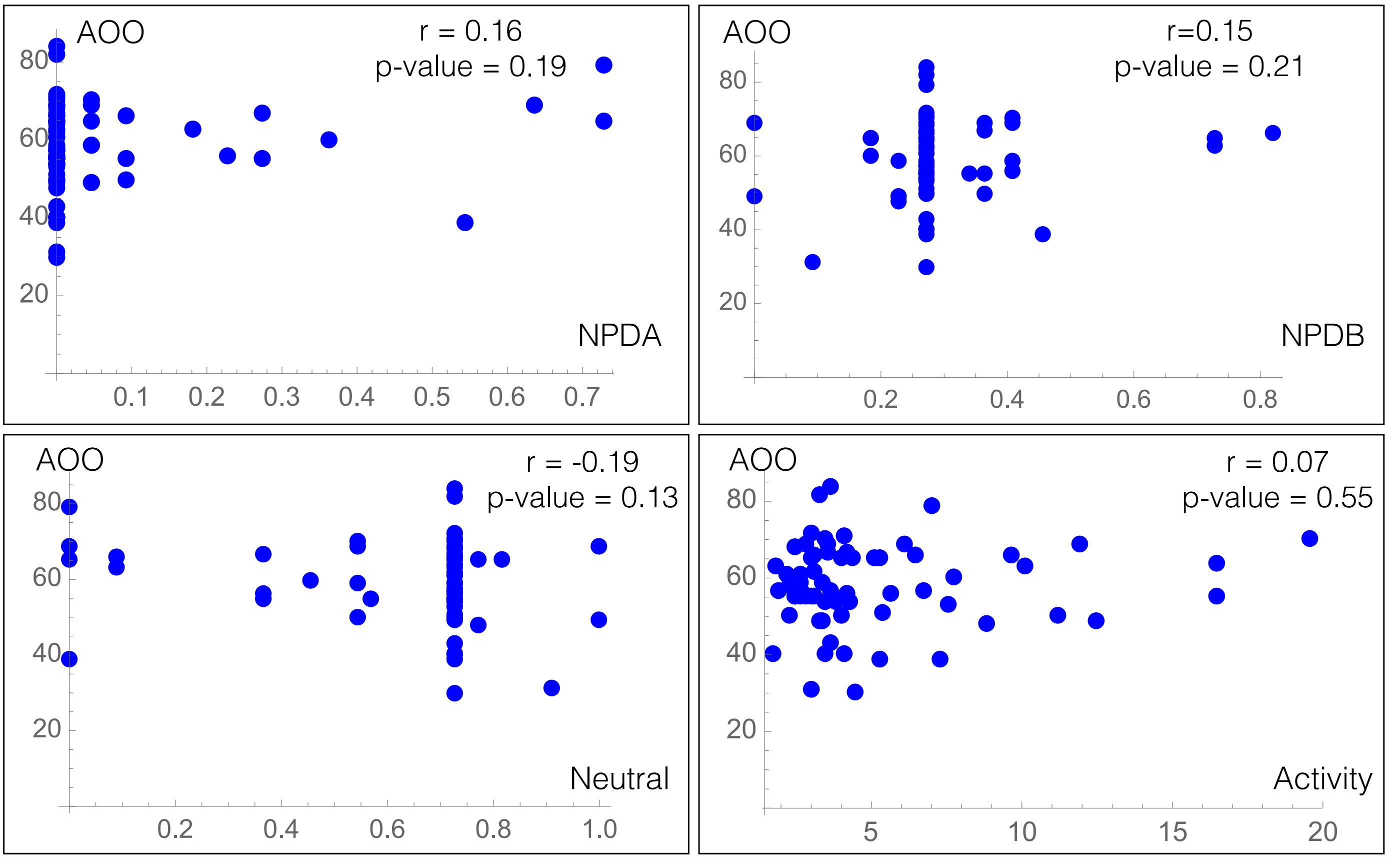


**Figure S4.** Plot of the age of onset of Parkinson disease (AOO) of individuals carrying rare SMPD1 variants as a function of the probabilities P(NPDA), P(NPDB) and P(Neutral) of these variants predicted by SMPD1-ZooM and of their activity measured in [9]. The linear correlations coefficients *r* and associated P-values are given.

## 10. Bibliography


1. Choi, Y.; Sims, G.E.; Murphy, S.; Miller, J.R.; Chan, A.P. Predicting the functional effect of amino acid substitutions and indels. *PloS one* **2012**, *7*, e46688. doi:10.1371/journal.pone.0046688.
2. Raimondi, D.; Tanyalcin, I.; Ferté, J.; Gazzo, A.; Orlando, G.; Lenaerts, T.; Rooman, M.; Vranken, W. DEOGEN2: prediction and interactive visualization of single amino acid variant deleteriousness in human proteins. *Nucleic acids research* **2017**, *45*, W201–W206. doi:10.1093/nar/gkx390.
3. Ancien, F.; Pucci, F.; Godfroid, M.; Rooman, M. Prediction and interpretation of deleterious coding variants in terms of protein structural stability. *Scientific Reports* **2018**, *8*, 1–11. doi:10.1038/s41598-018-22531-2.
4. Dehouck, Y.; Kwasigroch, J.M.; Gilis, D.; Rooman, M. PoPMuSiC 2.1: a web server for the estimation of protein stability changes upon mutation and sequence optimality. *BMC bioinformatics* **2011**, *12*, 151. doi:10.1186/1471-2105-12-151.
5. Cooper, G.M.; Hausman, R.E. *The cell : a molecular approach*, 5th ed. ed.; ASM Press: Washington, D.C, 2009; p. 53.
6. Xiong, Z.J.; Huang, J.; Poda, G.; Pomès, R.; Privé, G.G. Structure of Human Acid Sphingomyelinase Reveals the Role of the Saposin Domain in Activating Substrate Hydrolysis. *Journal of Molecular Biology* **2016**, *428*, 3026–3042. doi:10.1016/j.jmb.2016.06.012.
7. Consortium, T.U. UniProt: the universal protein knowledgebase. *Nucleic Acids Research* **2017**, *45*. doi:10.1093/nar/gkw1099.
8. Finn, R.D.; Coggill, P.; Eberhardt, R.Y.; Eddy, S.R.; Mistry, J.; Mitchell, A.L.; Potter, S.C.; Punta, M.; Qureshi, M.; Sangrador-Vegas, A.; Salazar, G.A.; Tate, J.; Bateman, A. The Pfam protein families database: Towards a more sustainable future. *Nucleic Acids Research* **2016**, *44*, D279–D285. doi:10.1093/nar/gkv1344.
9. Alcalay, R.N.; Mallett, V.; Vanderperre, B.; Tavassoly, O.; Dauvilliers, Y.; Wu, R.Y.; Ruskey, J.A.; Leblond, C.S.; Ambalavanan, A.; Laurent, S.B.; Spiegelman, D.; Dionne-Laporte, A.; Liong, C.; Levy, O.A.; Fahn, S.; Waters, C.; Kuo, S.H.; Chung, W.K.; Ford, B.; Marder, K.S.; Kang, U.J.; Hassin-Baer, S.; Greenbaum, L.; Trempe, J.F.; Wolf, P.; Oliva, P.; Zhang, X.K.; Clark, L.N.; Langlois, M.; Dion, P.A.; Fon, E.A.; Dupre, N.; Rouleau, G.A.; Gan-Or, Z. SMPD1 mutations, activity, and -synuclein accumulation in Parkinson's disease. *Movement Disorders* **2019**, *34*, 526–535. doi:10.1002/mds.27642.
